# Bayesian Covariance Structure Modeling of Responses and Process Data

**DOI:** 10.3389/fpsyg.2019.01675

**Published:** 2019-08-05

**Authors:** Konrad Klotzke, Jean-Paul Fox

**Affiliations:** Faculty of BMS, Department of Research Methodology, Measurement, and Data Analysis, University of Twente, Enschede, Netherlands

**Keywords:** process data, educational measurement, Bayesian modeling, covariance structure, marginal modeling, cross-classification, response times, latent variable modeling

## Abstract

A novel Bayesian modeling framework for response accuracy (RA), response times (RTs) and other process data is proposed. In a Bayesian covariance structure modeling approach, nested and crossed dependences within test-taker data (e.g., within a testlet, between RAs and RTs for an item) are explicitly modeled. The local dependences are modeled directly through covariance parameters in an additive covariance matrix. The inclusion of random effects (on person or group level) is not necessary, which allows constructing parsimonious models for responses and multiple types of process data. Bayesian Covariance Structure Models (BCSMs) are presented for various well-known dependence structures. Through truncated shifted inverse-gamma priors, closed-form expressions for the conditional posteriors of the covariance parameters are derived. The priors avoid boundary effects at zero, and ensure the positive definiteness of the additive covariance structure at any layer. Dependences of categorical outcome data are modeled through latent continuous variables. In a simulation study, a BCSM for RAs and RTs is compared to van der Linden's hierarchical model (LHM; van der Linden, 2007). Under the BCSM, the dependence structure is extended to allow variations in test-takers' working speed and ability and is estimated with a satisfying performance. Under the LHM, the assumption of local independence is violated, which results in a biased estimate of the variance of the ability distribution. Moreover, the BCSM provides insight in changes in the speed-accuracy trade-off. With an empirical example, the flexibility and relevance of the BCSM for complex dependence structures in a real-world setting are discussed.

## 1. Introduction

Computer-based assessments (CBAs) provide the opportunity to gather responses times (RTs) and other process data in addition to the test-takers' responses. Empirical research has shown that in combination with response patterns, RTs can lend valuable insight into interesting test-taker, item and test characteristics, such as pre-knowledge of items, motivation, time-pressure or differential speededness (Bridgeman and Cline, 2004; Wise and Kong, 2005; Meijer and Sotaridona, 2006; van der Linden et al., 2007; van der Linden and Guo, 2008; Marianti et al., 2014; Qian et al., 2016). New types of process data have been explored lately that carry the potential to lend additional insight into (latent) response processes and to improve inferences about constructs of interest (e.g., Azevedo, 2015; He et al., 2016; Goldhammer and Zehner, 2017; Maddox, 2017). To make valid inferences from process data, innovative joint models are needed that are capable of utilizing test-taker data beyond RAs and RTs, while accounting for complex relationships in multiple data types.

An important concept is the speed-accuracy trade-off, which states that, on average, a test-taker's ability suffers from an increased working speed (van der Linden, 2009). Test scores depend on test-takers' speed during the test and ignoring this within-subject relationship threatens the validity of inferences about their ability level. In experimental cognitive psychology, the speed-accuracy trade-off can be modeled for individual persons as the relationship between the proportion of correct tasks and the average time spent on the tasks (Luce, 1986). In educational measurement, learning effects can be expected when presenting the same item to a test-taker multiple times (Butler, 2010). Hence, in practical applications, often only a single measurement of RA and RT is obtained for each combination of test-taker and item. Therefore, it is common to assume a certain homogeneity in the speed-accuracy trade-off within a group of test-takers and how they are affected by the condition of interest (Thissen, 1983; Klein Entink et al., 2008; Glas and van der Linden, 2010; Ranger and Kuhn, 2013; Goldhammer and Kroehne, 2014; Goldhammer et al., 2014; Loeys et al., 2014; Molenaar et al., 2015; van der Linden and Fox, 2016). Alternatively, in certain experimental settings, the researcher can control the test-takers' working speed (by imposing time limits) and thereby exclude the person-level working speed variable from the regression equation (Goldhammer and Kroehne, 2014).

More general and flexible approaches to model and test the within-subject dependence structure have been achieved through the generalized linear mixed model (GLMM) (McCulloch, 2003) and mixture models. The within-subject mixture models allow subject-specific changes in the speed-accuracy trade-off across different states. However, in practice the number of states is very limited (Wang and Xu, 2015; Molenaar et al., 2016) to obtain identifiable and stable estimation results. In GLMMs, the measurement model for the RAs or the RTs is extended by including either the person level variable (ability or working speed) or the dependent variable of the respective other measurement model as a covariate in the regression equation. Item-specific person-level and person-specific item-level variables allow the speed-accuracy trade-off to vary between items and allow item parameters to vary across persons, respectively (e.g., Goldhammer et al., 2014, 2015). Furthermore, a non-linear relation between RAs and RTs can be specified (e.g., Molenaar et al., 2015; Bolsinova and Molenaar, 2018).

However, the complexity of a GLMM is drastically increased when including other process data and extending the GLMMs with additional person-level variables. It is therefore questionable whether the GLMM approach can manage the challenges of utilizing new types of process data in complex CBAs. Currently, GLMMs are limited in the amount of process data information that can be utilized to make inferences due to restrictions on the model complexity and the sample size. Furthermore, GLMMs are also limited in how the information is utilized. For instance, correlations between RAs and different types of process data may vary depending on item characteristics or test design. In that case, interaction effects are needed to model item and/or testlet-specific dependences, but this will significantly increase the complexity of the GLMM. To prevent over-parameterization and weak numerical stability, techniques such as principle component analysis, latent class analysis, or various model selection algorithms (e.g., backward elimination, forward selection or all subsets regression) (Thomas, 2002; Efron et al., 2004; Wetzel et al., 2015) have been proposed to reduce the number of covariates in the regression equation. However, this complicates a straightforward modeling approach and can lead to arbitrary assumptions and *ad hoc* decisions. It is well-known that ignoring correlations in test-taker data may cause violations of local independence assumptions and can result in biased inferences about parameters, the reliability of the test, and hinder test equating (e.g., Yen, 1984; Ackerman, 1987; Chen and Thissen, 1997; Bradlow et al., 1999; Baker and Kim, 2004; Jiao et al., 2005, 2012; Wang and Wilson, 2005; Wainer et al., 2007). Therefore, when including new types of process data, care must be taken in modeling the dependence structure to avoid making biased inferences.

The proposed Bayesian Covariance Structure Model (BCSM) can handle different types of nested and cross-classified dependence structures for multiple types of test-taker data. The BCSM extends the marginal model for hierarchically structured item RT data of Klotzke and Fox (2018). In the model of Klotzke and Fox (2018), dependences that follow from nested classifications (e.g., item clusters in a testlet design) are directly modeled as covariances without including random effects. The methodology is extended to classifications across multiple data types. Thus, in addition to modeling nested classifications (within a data type), relationships in data across different types (e.g., RTs and dichotomous responses) are modeled through cross-classifications in the dependence structure. In the same manner as the nested classifications, crossed classifications are modeled explicitly as covariance parameters. Without the inclusion of random effects, the parsimony of the BCSM is preserved, where dependences between each cluster of observations can be modeled with a single covariance parameter. The BCSM assumes a multivariate normal distribution for the data, either directly or through a threshold specification (i.e., for categorical or count data), and allows distinct modeling of the mean and covariance structure. The BCSM parameters can be estimated with an efficient Gibbs-sampling algorithm, even for a reasonably small sample size. Modeling local dependences via covariance parameters instead of modeling dependences through random effects (i.e., the random effect variance defines the covariance between clustered observations) has two advantages: first, covariances can be negative or positive, which allows more flexibility in specifying complex dependence structures than random effect variances. The latter can only model positive dependences. Second, tests for local independence under the BCSM framework do not require testing at the boundary of the parameter space (i.e., the null hypothesis states that the covariance parameter is equal to zero). This stands in contrast to a random effect variance, which is *a-priori* restricted to be positive. In the BCSM, this means that the prior distributions for the covariance parameters are less informative, i.e., they don't assume beforehand that the covariance parameters are greater than zero. Therefore, more objective inferences about the dependence structure can be made. Finally, contrary to common marginal modeling approaches such as generalized estimating equations (GEE) (Liang and Zeger, 1986; Diggle et al., 2013), the dependence structure is fully modeled in an additive covariance structure. This allows testing for interaction effects (e.g., local dependence within testlets) (Lee and Neider, 2004), and to estimate random person/group effects *post-hoc* from the residuals of the model. The latter is of utility if the random effects structure cannot be estimated in the traditional way (fitting a random effects model) due to for instance sample size limitations. For example, test-taker ability estimates can be obtained under a complex within-subject dependence structure, while accounting for various types of process data information.

The paper is organized as follows: first, the BCSM is introduced. Next, an additive covariance structure is defined that can be utilized to explicitly model dependences in data from different types (RAs, RTs, and other process data). Five well-known dependence structures are presented under the BCSM. An approach to model the interdependence of categorical data through truncated conditional univariate normal distributions of latent variables is specified. Closed-form expressions for the conditional posterior distributions of the covariance parameters are derived through truncated shifted inverse-gamma priors, where the truncation point ensures the positive definiteness of the additive covariance matrix. Samples from the full joint posterior are obtained using a Gibbs sampler. In a simulation study, a BCSM for RAs and RTs is compared to the hierarchical model of van der Linden (2007) (LHM) given a situation in which the test-taker's working speed and ability are allowed to vary over the course of the test, thus violating the assumption of local independence in models that presume a fixed working speed and ability. In an empirical example, data from the Programme for the International Assessment of Adult Competencies (PIAAC) study (OECD, 2013) is analyzed with a BCSM for RAs and two types of process data. Finally, the results, limitations and future prospects of the proposed framework for educational measurement applications are discussed.

## 2. Modeling Covariance Structures

Data can be dependent on different levels. For example, in a testlet structure data of items within a testlet may be correlated stronger than data across items. Hence, data may be locally dependent on a testlet level. Furthermore, more than one data point may be available for an item (e.g., dichotomous responses, RTs and additional process data). The relationship between two data points (of the same test-taker) for an item can be positive or negative. The corresponding local dependence can either increase (when positive) or decrease (when negative) the total correlation of data for an item. This multilevel dependence structure is specified through a cross-classification matrix. In BCSM, an additive covariance structure forms the link between the covariance parameters and the cross-classification matrix.

Observations within a group can be more alike than observations across groups. In the BCSM framework, this local dependence is modeled in an additive covariance structure. Each source of local dependence, i.e., the effect of each grouping on the association of components, is represented by a covariance parameter and a layer in the additive structure. Group membership is specified by a *N*_*t*_ × *N*_*c*_ classification matrix ***u***, where *N*_*t*_ is the number of layers in the additive covariance structure and *N*_*c*_ is the number of components. Each row in ***u*** thus corresponds to a layer in the covariance structure and the columns define the local dependence of components in that layer. Components that are grouped together within a layer are marked by a 1, ungrouped components are marked by a 0.

The components are assumed to be multivariate normally distributed with a *N*_*c*_-dimensional mean vector **μ** and a *N*_*c*_ × *N*_*c*_-dimensional covariance matrix **Σ**. The inclusion of person level random effects is not necessary as the covariance structure implied by the usual person level variables (such as ability and working speed) is directly modeled. As a consequence, the mean structure consists of intercepts on the group and item level. Intercepts on the group level are for example the average working speed or ability in a group of test-takers. Intercepts on the item level are commonly denoted as item time intensity and item difficulty parameters. Furthermore, test-taker background variables can be included as covariates.

The covariance matrix consists of a base layer **Σ**_0_ and *N*_*t*_ additive layers. In the base layer, the measurement error variance is modeled, whereby $\Sigma_{0}=diag\left(\sigma_{1}^{2},\ldots ,\sigma_{N_{c}}^{2}\right)$. Each additive layer *t* is constructed out of a covariance parameter θ_*t*_ and the *t*-th row of the classification vector, i.e., ***u***_*t*_:

(1) $$\begin{array}{r} \Sigma =\Sigma_{0}+\overset{N_{t}}{\underset{t=1}{\sum }}\theta_{t}u_{t}u_{t}^{T}. \end{array}$$

On a mathematical level, no qualitative difference is made between the covariance parameters **θ** = {δ, τ, ω, ϕ, **ν**, **Δ**}. However, for the sake of clarity, in this text δ, τ, and ω represent the covariance between, respectively, the RTs, the RAs and additional process data of a test-taker. The local dependence that follows from grouping observations from different data types on a person level is represented by the covariance parameter ϕ. The vector ***ν*** contains the cross-covariances between components of different types (e.g., RAs and RTs). Furthermore, **Δ** are the covariances that follow from blocks within components of one type (e.g., testlets within RT data).

Five examples of models for responses and process data are described that can be constructed within the BCSM framework. Each example is illustrated for a test size of *p* = 6 items. The utilized data types are RTs (***RT***), RAs that are manifested as discrete variables through a threshold specification (***RA***) and additional process data (***W***). The observed categorical data are denoted as ***D***. Finally, the scalability of BCSMs given a growing number of items and extensions to the classification structure is discussed.

### 2.1. The BCSM for Speed and Ability

The BCSM for speed and ability follows the classification as implied by the LHM with binary factor loadings. In this model, a test-taker's RTs are grouped by the latent factor working speed, and the RAs are grouped by the latent factor ability. Furthermore, observations are grouped across the two data types on a person level, which represents a correlation between a test-taker's ability and working speed. Table 1 shows the classification matrix and covariance parameters of the BCSM for speed and ability.

**Table 1:** The additive covariance structure of the BCSM for speed and ability is implied by the random effects structure of the LHM with binary factor loadings.

	**Classification matrix** *******u*******											
**Covariance**	**Response times**						**Response accuracies**					
δ	1	1	1	1	1	1	0	0	0	0	0	0
τ	0	0	0	0	0	0	1	1	1	1	1	1
ϕ	1	1	1	1	1	1	1	1	1	1	1	1

### 2.2. Variable Speed-Accuracy Trade-Off

**Figure 1:**
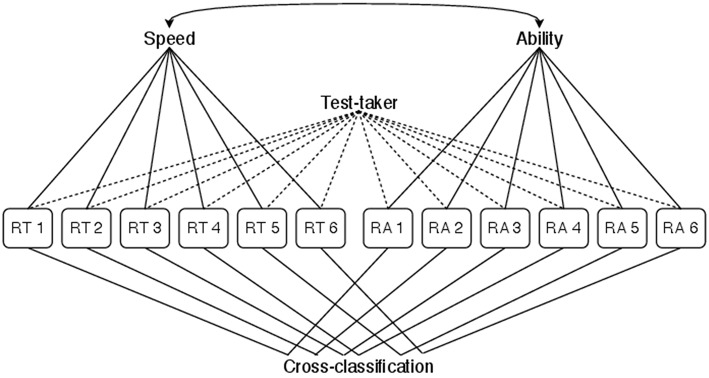
Classification diagram for the variable speed-accuracy trade-off model. The classification implied by the LHM is extended by grouping components item-wise. This allows the group level speed-accuracy trade-off to vary between items.

For the variable speed-accuracy trade-off model, the BCSM for speed and ability is extended with an item-specific cross-covariance between a test-taker's RTs and RAs. This allows to investigate how the speed-accuracy trade-off within a group of test-takers varies between items. Thereby, a certain homogeneity in the relevant response processes is assumed, which leads to test-takers within a group sharing a common speed-accuracy trade-off. The classification diagram for the variable speed-accuracy trade-off model is shown in Figure 1. Table 2 extends Table 1 with the additional classification rules and covariance parameters implied by a variable speed-accuracy trade-off.

**Table 2:** The additive covariance structure of the variable speed-accuracy trade-off model is an extension of the BCSM for speed and ability with item-specific cross-covariances between RTs and RAs.

	**Classification matrix** *******u*******											
**Covariance**	**Response times**						**Response accuracies**					
ν_1_	1	0	0	0	0	0	1	0	0	0	0	0
ν_2_	0	1	0	0	0	0	0	1	0	0	0	0
ν_3_	0	0	1	0	0	0	0	0	1	0	0	0
ν_4_	0	0	0	1	0	0	0	0	0	1	0	0
ν_5_	0	0	0	0	1	0	0	0	0	0	1	0
ν_6_	0	0	0	0	0	1	0	0	0	0	0	1

### 2.3. Blocked Structures of Cross-Covariances

Just as the variable speed-accuracy trade-off model, the blocked structures of cross-covariances model extends the BCSM for speed and ability with a varying cross-covariance between a test-taker's RTs and RAs. However, the cross-covariance is defined to change per blocks of (here: two) items. A possible application for this model is test-taking under varying time-pressure conditions. In such a scenario, it is reasonable to assume local dependence for components (i.e., RTs and RAs) within a block of items that belong to the same time-pressure condition. In the variable speed-accuracy trade-off model on the other hand, the local dependence is defined per individual item. Table 3 extends Table 1 with the additional classification rules and covariance parameters of the blocked structures of cross-covariances model.

**Table 3:** The additive covariance structure of the blocked structures of cross-covariances model is an extension of the BCSM for speed and ability with block-wise cross-covariances between RTs and RAs.

	**Classification matrix** *******u*******											
**Covariance**	**Response times**						**Response accuracies**					
ν_1_	1	1	0	0	0	0	1	1	0	0	0	0
ν_2_	0	0	1	1	0	0	0	0	1	1	0	0
ν_3_	0	0	0	0	1	1	0	0	0	0	1	1

### 2.4. Differential Blocked Structures of Cross-Covariances Across Factors

The within-subject dependence structure can also be specified for components within a single data type. In the differential blocked structures of cross-covariances across factors model, the variable speed-accuracy trade-off model is extended with a separate testlet structure for each the RTs and the RAs. The testlet structures are defined independently of each other. Table 4 extends Tables 1, 2 with the additional classification rules and covariance parameters of the differential blocked structures of cross-covariances across factors model.

**Table 4:** The additive covariance structure of the differential blocked structures of cross-covariances across factors model is an extension of the variable speed-accuracy trade-off model with independent testlet structures for separate data types.

	**Classification matrix** *******u*******											
**Covariance**	**Response times**						**Response accuracies**					
Δ_1_	1	1	0	0	0	0	0	0	0	0	0	0
Δ_2_	0	0	1	1	0	0	0	0	0	0	0	0
Δ_3_	0	0	0	0	1	1	0	0	0	0	0	0
Δ_4_	0	0	0	0	0	0	1	1	1	0	0	0
Δ_5_	0	0	0	0	0	0	0	0	0	1	1	1

### 2.5. More Than Two Data Types

A BCSM is not limited to RTs and responses. Additional process data can carry information relevant to the research. In this example, additional process data is available for each combination of test-taker and item. Therefore, *p* = 6 components are added to the model. In the illustrated model, an item-specific cross-covariance between components of all types is assumed. That means for example that RTs and RAs to an item may correlate in a different way than RAs and process data, to the same item. Furthermore, ϕ_1_, ϕ_2_, and ϕ_3_ represent the 3-by-3 covariance of the three latent factors (e.g., ability, working speed, and speed first action) that are related to the three types of data. Table 5 shows the classification matrix and covariance parameters of the more than two data types model.

**Table 5:** The additive covariance structure for a BCSM that incorporates additional process data, next to RTs and RAs.

	**Classification matrix** *******u*******																	
**Covariance**	**Response times**						**Response accuracies**						**Process data**					
δ	1	1	1	1	1	1	0	0	0	0	0	0	0	0	0	0	0	0
τ	0	0	0	0	0	0	1	1	1	1	1	1	0	0	0	0	0	0
ω	0	0	0	0	0	0	0	0	0	0	0	0	1	1	1	1	1	1
ϕ_1_	1	1	1	1	1	1	1	1	1	1	1	1	0	0	0	0	0	0
ϕ_2_	1	1	1	1	1	1	0	0	0	0	0	0	1	1	1	1	1	1
ϕ_3_	0	0	0	0	0	0	1	1	1	1	1	1	1	1	1	1	1	1
ν_1_	1	0	0	0	0	0	1	0	0	0	0	0	0	0	0	0	0	0
ν_2_	0	1	0	0	0	0	0	1	0	0	0	0	0	0	0	0	0	0
ν_3_	0	0	1	0	0	0	0	0	1	0	0	0	0	0	0	0	0	0
ν_4_	0	0	0	1	0	0	0	0	0	1	0	0	0	0	0	0	0	0
ν_5_	0	0	0	0	1	0	0	0	0	0	1	0	0	0	0	0	0	0
ν_6_	0	0	0	0	0	1	0	0	0	0	0	1	0	0	0	0	0	0
ν_7_	1	0	0	0	0	0	0	0	0	0	0	0	1	0	0	0	0	0
ν_8_	0	1	0	0	0	0	0	0	0	0	0	0	0	1	0	0	0	0
ν_9_	0	0	1	0	0	0	0	0	0	0	0	0	0	0	1	0	0	0
ν_10_	0	0	0	1	0	0	0	0	0	0	0	0	0	0	0	1	0	0
ν_11_	0	0	0	0	1	0	0	0	0	0	0	0	0	0	0	0	1	0
ν_12_	0	0	0	0	0	1	0	0	0	0	0	0	0	0	0	0	0	1
ν_13_	0	0	0	0	0	0	1	0	0	0	0	0	1	0	0	0	0	0
ν_14_	0	0	0	0	0	0	0	1	0	0	0	0	0	1	0	0	0	0
ν_15_	0	0	0	0	0	0	0	0	1	0	0	0	0	0	1	0	0	0
ν_16_	0	0	0	0	0	0	0	0	0	1	0	0	0	0	0	1	0	0
ν_17_	0	0	0	0	0	0	0	0	0	0	1	0	0	0	0	0	1	0
ν_18_	0	0	0	0	0	0	0	0	0	0	0	1	0	0	0	0	0	1

### 2.6. Model Scalability

The models constructed in the BCSM framework are scalable with respect to the length of the test, the number of data types and the specified dependence structure. The number of columns of ***u*** corresponds to the number of data components (*N*_*c*_). If a single observation is available for each combination of test-taker, item and data type, the number of data components is the product of the number of items (*p*) and the number of data types (*N*_*d*_), i.e., *N*_*c*_ = *p* * *N*_*d*_. Consequently, extending the test length with one item increases the number of columns of ***u*** by *N*_*d*_. Similarly, introducing an additional data type increases the number of columns by *p*.

The number, if any, of additional rows of ***u*** depends on the specified classification structure. For example, under the structure specified in Table 1, a change in the number of data components does not affect the number of rows of ***u***. Instead, the existing groupings are extended to include the new data components.

In other situations, the number of groupings depends on the number of data components. For example, given the item-specific cross-classifications as defined in Table 2, each additional item leads to one additional classification rule (the RA and RT of a test-taker to one item are grouped together) and therefore inserts one row into ***u***. Thus, if the variable speed-accuracy trade-off joint-model is applied to a test with *p*_2_ = 100 instead of *p*_1_ = 10 items, the number of columns increases by (*p*_2_−*p*_1_) * *N*_*d*_ = (100−10) * 2 = 180 and the number of rows increases by *p*_2_−*p*_1_ = 90.

## 3. Categorical Outcome Data

When recording the test-takers' responses during a test, discrete realizations of latent response variables are observed. The multivariate normally distributed RA data (latent responses) are linked through a threshold specification to their discrete realizations. However, truncating a multivariate normal distribution in high dimensions is non-trivial (Botev, 2017) and simply truncating independently for each dimension does not lead to the intended multivariate joint-distribution (Horrace, 2005).

The proposed solution is to derive the univariate normal distribution of each latent response component, conditional on all other components. The univariate normal distribution is derived by partitioning the additive covariance structure **Σ**, as defined in Equation 1, into four parts. The upper left part, ***B***_11_, gives the variance of the *k*-th component and the diagonal parts, ***B***_12_ and ***B***_21_, contain the covariance of the *k*-th component with the remaining components. Finally, ***B***_22_ describes the covariance structure of all components but the *k*-th:

(2) $$\begin{array}{l} \begin{array}{cc} \;Y_{ik} & {\tilde{\;Y}}_{i} \end{array}\\ \;\Sigma =\left[\begin{array}{ll} B_{11} & B_{12}\\ B_{21} & B_{22} \end{array}\right]\begin{array}{l} Y_{ik}\\ {\tilde{Y}}_{i} \end{array}, \end{array}$$

where ***Y*** is a *N* × *N*_*c*_-dimensional matrix, containing data from all *N*_*c*_ components and *N* test-takers. A tilde, i.e., a ~, above a vector or matrix indicates that the *k*-th component is excluded from the data structure. Based on the partitioned covariance matrix, the means and variance of the conditionally univariate normal distribution of the *k*-th component are derived for each test-taker:

(3) $$\begin{array}{rc} \mu_{Y_{k}|\tilde{Y}} & =\mu_{Y_{k}}+B_{12}B_{22}^{-1}\left(\tilde{Y}-\mu_{Ỹ}\right), \end{array}$$

(4) $$\begin{array}{rc} \sigma_{Y_{k}|\tilde{Y}}^{2} & =B_{11}-B_{12}B_{22}^{-1}B_{21}. \end{array}$$

A closed-form expression for $B_{22}^{-1}$ is derived through the Sherman-Morrison formula (e.g., Lange, 2010, p. 261):

(5) $$\begin{array}{r} A_{t+1}^{-1}={\left(A_{t}+\lambda vv^{T}\right)}^{-1}=A_{t}^{-1}-\frac{A_{t}^{-1}vv^{T}A_{t}^{-1}}{1/\lambda +v^{T}A_{t}^{-1}v}, \end{array}$$

where $A_{t}^{-1}={\tilde{\Sigma }}_{t}^{-1}$ is the inverse of the additive covariance structure for all but the *k*-th component at the *t*-th layer, λ = θ_*t*+1_ is the covariance parameter for the added layer and $v={\tilde{u}}_{t+1}$ contains the classification structure for the new layer. Given that the inverse of $A_{0}^{-1}={\tilde{\Sigma }}_{0}^{-1}$, i.e., the inverse of the diagonal matrix consisting of the measurement error variance parameters for all but the *k*-th component, is known, the inverse for any additional layer can be derived recursively.

## 4. Bayesian Inference

In line with the approach suggested by Fox et al. (2017) and Klotzke and Fox (2018), closed-form expressions for the conditional posterior distributions of the variance and covariance parameters are derived through truncated shifted inverse-gamma priors. For each of the *N*_*t*_ layers of the additive covariance matrix, a truncation point *tr*_*t*_ is derived by applying the Sherman-Morrison formula (Lange, 2010, p. 260–261). Enforcing the truncation through the indicator function 𝟙_*tr*_ ensures that the covariance matrix is positive definite at any layer *t*. This leads to a lower bound for each covariance parameter (θ_*t*_ > *tr*_*t*_) conditional on the classification structure and the inverse of the covariance matrix at the underlying layer (*t* − 1):

(6) $$\begin{array}{r} tr_{t}=-1/u_{t}^{T}\Sigma_{t-1}^{-1}u_{t}. \end{array}$$

For the measurement error variance parameters (the diagonal terms of **Σ**_0_) a truncation sets the probability of negative values a-priori to zero.

The reasoning behind the shift parameters is based upon two premises: (1) a draw of θ_*t*_ is obtained through sampling θ_*t*_ + ψ_*t*_ and subtracting the shift parameter ψ_*t*_ iteratively within the Markov chain Monte Carlo (MCMC) (Gilks et al., 1995) algorithm, (2) the probability distribution of θ_*t*_ + ψ_*t*_ must incorporate all information that is available in the data about θ_*t*_. It is shown in Equations (7) and (8) that the probability distribution of the person level means across that are grouped together in ***u***_*t*_ contains all available information about the covariance parameter θ_*t*_. Note that the person level means are constructed as the mean of (correlated) random normal variables and are therefore univariate normally distributed.

Conditional on the classification structure and the additive covariance matrix at its highest layer (**Σ**_*N*_*t*__), the variance of the person level means is derived through the property that the variance of the sum of correlated random variables is the sum of their covariances:

(7) $$\begin{array}{l} Var({\bar{Y}}_{i(k\in u_{t})}|\Sigma_{N_{t}},u)=Var\left(\sum_{k\in u_{t}}Y_{ik}/(1_{N_{c}}^{T}u_{t})\right)\\ \;=[(\sum_{k=1}^{N_{c}}\sigma_{k}^{2}u_{tk}+\theta_{t}{\left(1_{N_{c}}^{T}u_{t}\right)}^{2}\\ \;+\sum_{j\neq t}\theta_{j}{\left(1_{N_{c}}^{T}(u_{j}⊙u_{t})\right)}^{2})/{\left(1_{N_{c}}^{T}u_{t}\right)}^{2}]\\ \;=\theta_{t}+\left[\left(\sum_{k=1}^{N_{c}}\sigma_{k}^{2}u_{tk}+\sum_{j\neq t}\theta_{j}{\left(1_{N_{c}}^{T}(u_{j}⊙u_{t})\right)}^{2}\right)/{\left(1_{N_{c}}^{T}u_{t}\right)}^{2}\right]\\ \;=\theta_{t}+\psi_{t}, \end{array}$$

where ⊙ denotes the Hadamard product and **1**_*N*_*c*__ is a *N*_*c*_-dimensional vector of ones. A sufficient statistic for *Var* (*Ȳ*_*i*(*k*∈_***u***__*t*_)_|**Σ**_*N*_*t*__, ***u***) = θ_*t*_ + ψ_*t*_ is therefore the sum of squares of the deviations of the conditional person level means from the conditional grand mean,

(8) $$\begin{array}{rc} SSB_{t} & =\overset{N}{\underset{i=1}{\sum }}{\left({\bar{Y}}_{i\left(k\in u_{t}\right)}-{\bar{Y}}_{.\left(k\in u_{t}\right)}\right)}^{2}. \end{array}$$

Similarly, the within-component sum of squares is a sufficient statistic for $Var\left(Y_{ik}\right)=\sigma_{k}^{2}+\overset{N_{t}}{\underset{t=1}{\sum }}\theta_{t}u_{tk}$, namely

(9) $$\begin{array}{rc} SSW_{k} & =\overset{N}{\underset{i=1}{\sum }}{\left(Y_{ik}-{\bar{Y}}_{.k}\right)}^{2}. \end{array}$$

From Equations (8) and (9) follow *N*_*t*_ + *N*_*c*_ sufficient statistics for the *N*_*t*_ covariance and *N*_*c*_ variance parameters, out of which the additive covariance structure, as specified in Equation (1), is composed. The model is therefore identified under the condition that the rows of the classification matrix ***u*** are mutually distinct.

The truncated shifted inverse-gamma prior extends the default inverse-gamma prior for variance components with a shift and a truncation parameter; the former allowing a covariance parameter to take on negative values, the latter ensuring the positive definiteness of the additive covariance matrix at any layer:

(10) $$\begin{array}{l} IG\left(x,\alpha_{0},\beta_{0},\psi_{t},tr_{t}\right)=\left[\frac{\beta_{0}^{\alpha_{0}}}{\Gamma \left(\alpha_{0}\right)}{\left(x+\psi_{t}\right)}^{-\alpha_{0}-1}exp\left(-\frac{\beta_{0}}{x+\psi_{t}}\right)\right]\cdot \\ \;{𝟙}_{tr}\left(x>tr_{t}\right), \end{array}$$

where the truncation point (*tr*_*t*_) and shift parameter (ψ_*t*_) are computed according to Equations (6) and (7).

Note that conjugacy between the extended inverse-gamma prior and the likelihood function of a normal distribution is preserved, thus leading to truncated shifted inverse-gamma posteriors for the covariance and measurement error variance parameters:

(11) $$\begin{array}{rc} \theta_{t} & \sim IG\left(x,\alpha_{0}+N/2,\beta_{0}+SSB_{t}/2,\psi_{t},tr_{t}\right), \end{array}$$

(12) $$\begin{array}{rc} \sigma_{k}^{2} & \sim IG\left(x,\alpha_{0}+N/2,\beta_{0}+SSW_{k}/2,\overset{N_{t}}{\underset{t=1}{\sum }}\theta_{t}u_{tk},0\right). \end{array}$$

The a-priori restriction of $\sigma_{k}^{2}>0$ is thus enforced by fixing the truncation point for the measurement error variance parameters to zero.

See Appendix A in Supplementary Material for an outline of the MCMC algorithm and the corresponding sampling steps.

## 5. Simulation Study

In a simulation study, the within-subject dependence structure under a model for RTs and dichotomous responses is estimated. A comparison is made between a BCSM and the LHM. In the BCSM framework, the dependence structure is directly modeled in an additive covariance matrix. In the LHM framework, the dependence structure is implied by the random effect structure and in particular the random effect variances. Therefore, the focus of this simulation study is the precision and bias of the (co)variance parameter estimates.

In the simulated experiment, across two conditions, *N* = 200 and *N* = 1, 000 randomly selected persons are taking a test that consists of *p* = 12 items. Furthermore, the time-pressure on the test-takers systematically changes after every two items. This is assumed to affect the response processes within the group of test-takers over the course of the test. For example, under a perceived high time-pressure, guessing may become more likely. The change in response processes is reflected by the within-subject dependence structure, i.e., the speed-accuracy trade-off may vary between blocks of two items and is common across test-takers.

The length of the test is fixed across the 100 replications for both conditions of the simulation. Within each condition, all test-takers are part of the same group. Within each replication, test-taker data are generated and the BCSM as well as the LHM are fitted with 5000 MCMC iterations and a burn-in phase of 10%. The LHM is fitted using the R-package LNIRT (Fox et al., 2018).

### 5.1. LHM for Fixed Speed and Ability

On the first level of the hierarchical framework, separate measurement models for the RTs and RAs are specified. The item discrimination parameters are fixed to 1, which gives the following first level models for the RTs (*RT*) and RAs (*RA*) of test-taker *i* and item *k*:

(13) $$\begin{array}{rc} RT_{ik} & =\beta_{k}-\zeta_{i}+e_{RT_{ik}}, \end{array}$$

(14) $$\begin{array}{rc} RA_{ik} & =\theta_{i}-b_{k}+e_{RA_{ik}}, \end{array}$$

where $\zeta_{i}\sim N\left(\mu_{\zeta },\delta \right)$ and $\theta_{i}\sim N\left(\mu_{\theta },\tau \right)$ are random variables on a person level, representing the variation in working speed and ability between test-takers. The time intensity and item difficulty parameters β_*k*_ and *b*_*k*_ are item level intercepts and are not given further attention in this simulation study. Finally, $e_{RT_{ik}}\sim N\left(0,\sigma_{k}^{2}\right)$ and $e_{RA_{ik}}\sim N\left(0,1\right)$ are the measurement errors. On the second level, a model for the joint-distribution of the person parameters (working speed and ability) is defined:

(15) $$\begin{array}{r} \Sigma_{p}=\left(\begin{array}{cc} \delta +\phi & \phi \\ \phi & \tau +\phi \end{array}\right). \end{array}$$

Note that the LHM assumes a constant working speed and ability across the test for a test-taker. From this follows a test-wide cross-covariance between a test-taker's RTs and RAs ϕ.

### 5.2. BCSM for Variable Speed and Ability

In the BCSM, the within-subject dependence structure is modeled directly in an additive covariance structure with 9 layers. The covariance structure is defined in Equation (1), where **θ** = {δ, τ, ϕ, ν_1_, …, ν_6_} are the (cross-)covariance parameters and the classification matrix is specified in Table 6. A truncated shifted inverse-gamma prior with *shape* = 10^−3^ and *scale* = 10^3^ is defined for the variance and covariance parameters.

**Table 6:** The additive covariance structure of the BCSM allows a varying speed-accuracy trade-off between blocks of two items.

	**Classification matrix** *******u*******																							
**Covariance**	**Response times**												**Response accuracies**											
δ	1	1	1	1	1	1	1	1	1	1	1	1	0	0	0	0	0	0	0	0	0	0	0	0
τ	0	0	0	0	0	0	0	0	0	0	0	0	1	1	1	1	1	1	1	1	1	1	1	1
ϕ	1	1	1	1	1	1	1	1	1	1	1	1	1	1	1	1	1	1	1	1	1	1	1	1
ν_1_	1	1	0	0	0	0	0	0	0	0	0	0	1	1	0	0	0	0	0	0	0	0	0	0
ν_2_	0	0	1	1	0	0	0	0	0	0	0	0	0	0	1	1	0	0	0	0	0	0	0	0
ν_3_	0	0	0	0	1	1	0	0	0	0	0	0	0	0	0	0	1	1	0	0	0	0	0	0
ν_4_	0	0	0	0	0	0	1	1	0	0	0	0	0	0	0	0	0	0	1	1	0	0	0	0
ν_5_	0	0	0	0	0	0	0	0	1	1	0	0	0	0	0	0	0	0	0	0	1	1	0	0
ν_6_	0	0	0	0	0	0	0	0	0	0	1	1	0	0	0	0	0	0	0	0	0	0	1	1

### 5.3. Data Generation

Data are generated under a generalization of the models specified in Equations (13)–(15) that allows the test-takers' working speed and ability to vary over the course of the test:

(16) $$\begin{array}{rc} RT_{ik} & =\beta_{k}-\zeta_{it\left(k\right)}+e_{RT_{ik}}, \end{array}$$

(17) $$\begin{array}{rc} RA_{ik} & =\theta_{it\left(k\right)}-b_{k}+e_{RA_{ik}}. \end{array}$$

(18) $$\begin{array}{rc} \Sigma_{pk} & =\left(\begin{array}{cc} \delta +\phi +\nu_{t\left(k\right)} & \phi +\nu_{t\left(k\right)}\\ \phi +\nu_{t\left(k\right)} & \tau +\phi +\nu_{t\left(k\right)} \end{array}\right), \end{array}$$

where *t*(*k*) denotes item *k* in classification group *t*. The population values of the (co)variance parameters are δ = 0.5, τ = 0.5, ϕ = 0.5 and ***ν*** = {0, −0.05, −0.1, 0.4, 0.2, 0.3}. The item level intercepts (**β** and ***b***) are set to zero. Finally, the population values of the measurement error variances are generated from a uniform distribution with lower bound 0.5 and upper bound 1.5.

### 5.4. Results

Under the LHM, the test-wide cross-covariance and the variance of the test-taker working speed distribution are successfully estimated. The variance of the ability distribution (τ) is underestimated for both sample size conditions under the LHM, which can be attributed to ignoring the block-wise deviations from the test-wide cross-covariance. Under the BCSM, the full within-subject dependence structure is successfully estimated. Cross-covariances near zero (ν_1_, ν_2_, and ν_3_) are estimated without bias regardless of sample size, which can be attributed to the non-informative truncated shifted inverse-gamma priors. The standard deviations of the posterior mean estimates are comparable for both models. Increasing the sample size leads to smaller standard deviations of the posterior mean estimates for both models. Under the BCSM, an average correlation of 0.99 (SD: 0.01) is observed under both conditions between the simulated measurement error variance parameters and their posterior mean estimates. The results of the simulation study are summarized in Table 7.

**Table 7:** Means and standard deviations of posterior mean estimates across 100 simulated replications of data for 200 and 1,000 test-takers and 12 items.

	**Mean (SD) of posterior mean estimates**			
	***N*** **= 200**		***N*** **= 1,000**	
**Cov**	**BCSM**	**LHM**	**BCSM**	**LHM**
δ = 0.5	0.49 (0.03)	0.51 (0.03)	0.50 (0.01)	0.52 (0.01)
τ = 0.5	0.51 (0.07)	0.45 (0.06)	0.48 (0.03)	0.42 (0.03)
ϕ = 0.5	0.51 (0.03)	0.50 (0.03)	0.50 (0.02)	0.49 (0.01)
ν_1_ = 0	0.00 (0.04)		−0.01 (0.02)	
ν_2_ = −0.05	−0.05 (0.04)		−0.06 (0.03)	
ν_3_ = −0.1	−0.07 (0.03)		−0.11 (0.03)	
ν_4_ = 0.4	0.39 (0.06)		0.39 (0.03)	
ν_5_ = 0.2	0.18 (0.05)		0.19 (0.02)	
ν_6_ = 0.3	0.28 (0.06)		0.30 (0.02)	

*A comparison is made between a BCSM and the LHM. In the BCSM framework, the full within-subject dependence structure is modeled*.

## 6. Empirical Example: PIAAC 2012

The Programme for the International Assessment of Adult Competencies (PIAAC) study deploys a computer-based large scale assessment to gain insight into adult competencies across the domains of numeracy, literacy and problem solving (OECD, 2013). The computer-based nature of the assessment allows recording behavioral process data, in addition to the scored responses. It is assumed that the process data correlate with the scored responses and therefore contain information about the latent competencies of interest. Describing these correlations requires paying attention to local dependences within the data. Local dependences follow from shared item characteristics (e.g., response mode), the test design (e.g., testlets), the manner the process data is obtained (e.g., a single measurement per type, test-taker and item, multiple measurements or aggregated data) and the latent factor structure (e.g., data components load on test-takers' ability and working speed). Furthermore, test-taker characteristics such as computer experience or gender may affect the associations of data components (e.g., the correlation of RTs and RAs of an item may differ between test-takers with and without computer experience). It will be shown that a BCSM can be constructed that (a) takes the complex dependence structure within test-taker data into account, (b) allows correcting for between-subject differences in the dependence structure by including test-taker background variables, and (c) can be estimated given a reasonable sample size.

### 6.1. Data Set

The data set consists of responses and process data for *N* = 745 Canadian test-takers and *p* = 15 items. For each combination of item and test-taker, three data points are available: the scored dichotomous response, the total (log) RT it took the test-taker to complete the item and the (log) time it took the test-taker until they took their first action on that item. Nine of the items measure numeracy competencies, the remaining six items measure literacy competencies. Furthermore, the items differ in their response mode. See Table 8 for an overview of the included items and their characteristics. Moreover, the test-takers' gender (0: male, 1: female), computer experience (0: no, 1: yes), whether or not they are a native speaker (0: no, 1: yes) and their educational level (1: low, 2: medium, 3: high) were recorded. Further information on test-taker demographics and item characteristics can be found in Statistics Canada (2013).

**Table 8:** Id, name, domain, and response mode of the 15 PIAAC items included in the data analysis of the empirical example.

**Item id**.	**Name**	**Domain**	**Response mode**
1	Wine 1	Numeracy	Number match
2	Wine 2	Numeracy	Stimulus clicking
3	Gas gauge	Numeracy	Number match
4	Photo 1	Numeracy	Number match
5	Photo 2	Numeracy	Stimulus clicking
6	Photo 3	Numeracy	Exact match
7	Urban population	Numeracy	Number match
8	Tiles	Numeracy	Exact match
9	Package	Numeracy	Stimulus clicking
10	Baltic stock market 1	Literacy	Stimulus clicking
11	Baltic stock market 2	Literacy	Stimulus highlighting
12	Baltic stock market 3	Literacy	Stimulus clicking
13	Baltic stock market 4	Literacy	Stimulus clicking
14	TMN antitheft 1	Literacy	Stimulus highlighting
15	TMN antitheft 2	Literacy	Stimulus highlighting

### 6.2. Dependence Structure

**Figure 2:**
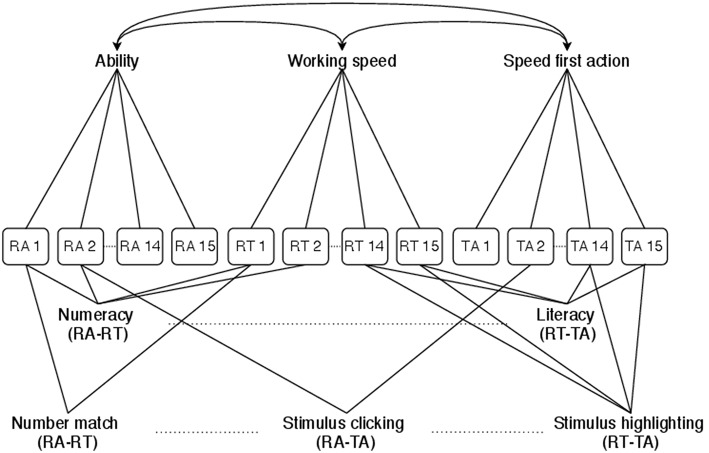
Classification diagram for the PIAAC 2012 BCSM. The classification structure specifies dependences between scored responses and behavioral process data for varying item characteristics (domain and response mode) and a correlated latent factors structure. RA, RAs that underlie the scored dichotomous responses; RT, total RTs per item; TA, times to first action per item.

Data that are naturally grouped may be stronger correlated than (conditionally) unrelated data. In the data set at hand, items are grouped through their domain (numeracy or literacy) and their response mode (number match, exact match, stimulus clicking or stimulus highlighting). For each grouping, three layers are defined: one for each pair of data types. This allows to explore how the dependences between, respectively, RAs and RTs, RAs and times to first action (TAs), and RTs and TAs vary across item domains and response modes, while controlling for the rest of the dependence structure. Furthermore, data components that load on a common latent factor may be correlated. Latent factors are the test-taker's ability, working speed and speed first action. The correlation between the latent factors is modeled in separate layers. Figure 2 illustrates the classifications that follow from the groupings. Data within each classification group may be locally dependent. The corresponding classification matrix for the *N*_*c*_ = 45 data components and *N*_*t*_ = 24 classification groups is shown in Appendix B (Supplementary Material).

### 6.3. Statistical Model

Under the BCSM framework, a model for response and process data is constructed. In the mean structure of the joint-model, test-taker background data are modeled as predictor variables. The dependence structure is modeled through an additive covariance matrix that defines the relationship of the multivariate normally distributed error terms:

(19) $$\begin{array}{r} Y_{i}=X_{i}B+\varepsilon_{i},\varepsilon_{i}\sim N\left(0_{N_{c}},\Sigma \right), \end{array}$$

where ***Y*** = {***RA***, ***RT***, ***TA***} is a *N* × *N*_*c*_-dimensional matrix containing the RAs that underlie the scored dichotomous responses (RA), the total RTs per item for each test-taker (RT), and the time passed until the test-taker's first action per item (TA). The *N* × 5-dimensional matrix ***X*** contains the grand-mean centered test-taker background variables (gender, computer experience, native speaker and education level) and a vector of ones as first column. ***B*** is a 5 × *N*_*c*_ matrix containing the regression weights for each of the four covariates on the *N*_*c*_ data components, and the intercepts. The first column of ***B*** contains the item-specific intercepts, which can be interpreted as item difficulty, time intensity and average time to first action parameters. The weights and intercepts are thus modeled for each data component and are equal across test-takers, therefore representing fixed effects. Note that no random variance components are associated with fixed effects, whereby they don't enter the modeled dependence structure. The *N*_*c*_ × *N*_*c*_-dimensional additive covariance matrix **Σ** consists of *N*_*t*_ = 24 layers that correspond to the specified dependence structure:

(20) $$\begin{array}{r} \Sigma =diag\left(\sigma \right)+\overset{N_{t}}{\underset{t=1}{\sum }}\theta_{t}u_{t}u_{t}^{T}. \end{array}$$

For the RA components, the measurement error variance parameters are fixed to one. Furthermore the scale of the IRT model is set by fixing the mean of the item-specific intercepts (i.e., the mean of the item difficulty parameters) to zero. The classification matrix ***u*** is shown in Appendix B (Supplementary Material). A truncated shifted inverse-gamma prior with *shape* = 10^−3^ and *scale* = 10^3^ is defined for the variance and covariance parameters. No a-priori information about the regression weights is used: the prior guesses for the scale matrix and the mean matrix of ***B*** equal the identity matrix and a matrix of zeros, respectively.

### 6.4. Results

**Figure 3:**
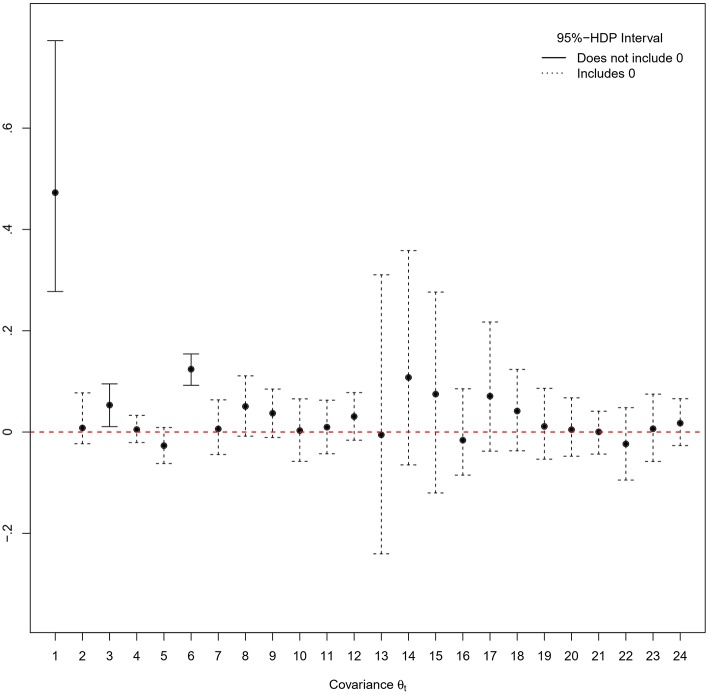
95%-Highest Posterior Density (HPD) intervals for the *N*_*t*_ = 24 covariance parameters in the additive covariance structure. Black dots correspond to posterior mean estimates.

The model parameters are estimated with a single MCMC chain of 55,000 iterations from which the first 15,000 iterations are discarded as burn-in period. Visual inspection of traceplots and applying the Heidelberger and Welch' criterion (Heidelberger and Welch, 1983) using the R-package coda (Plummer et al., 2016) indicate a satisfying exploration of the parameter space and do not provide evidence against convergence of the MCMC algorithm. The posterior means and standard deviations of the twenty-four covariance parameters in the additive covariance structure are summarized in Table 9. Figure 3 shows the corresponding 95%-Highest Posterior Density (HPD) intervals.

**Table 9:** Posterior means and standard deviations of the *N*_*t*_ = 24 covariance parameters in the additive covariance structure.

**Layer**	**Classification**	**Level**	**Posterior distribution**	
			**Mean**	**SD**
1	Ability	Latent factor	0.47	0.16
2	Working speed	Latent factor	0.01	0.03
3	Speed first action	Latent factor	0.05	0.02
4	Ability-Working speed	Latent factor	0.01	0.01
5	Ability-Speed first action	Latent factor	−0.03	0.02
6	Working speed-Speed first action	Latent factor	0.12	0.02
7	Numeracy: RA-RT	Item domain	0.01	0.03
8	Numeracy: RA-TA	Item domain	0.05	0.03
9	Numeracy: RT-TA	Item domain	0.04	0.02
10	Literacy: RA-RT	Item domain	0.00	0.03
11	Literacy: RA-TA	Item domain	0.01	0.03
12	Literacy: RT-TA	Item domain	0.03	0.02
13	Exact match: RA-RT	Response mode	−0.01	0.17
14	Exact match: RA-TA	Response mode	0.11	0.11
15	Exact match: RT-TA	Response mode	0.07	0.10
16	Number match: RA-RT	Response mode	−0.02	0.05
17	Number match: RA-TA	Response mode	0.07	0.07
18	Number match: RT-TA	Response mode	0.04	0.04
19	Stimulus clicking: RA-RT	Response mode	0.01	0.04
20	Stimulus clicking: RA-TA	Response mode	0.00	0.03
21	Stimulus clicking: RT-TA	Response mode	0.00	0.02
22	Stimulus highlighting: RA-RT	Response mode	−0.02	0.04
23	Stimulus highlighting: RA-TA	Response mode	0.01	0.03
24	Stimulus highlighting: RT-TA	Response mode	0.02	0.02

*Each layer of the covariance structure corresponds to one classification. Classifications are made across three data types (RA, response accuracies that underlie the scored dichotomous responses; RTs, response times; TAs, times to first action taken) based on (correlated) latent factors, item domains, and item response modes*.

Given the observed data, it can be concluded that the probability of local dependence in the ability and speed first action latent factor classification groups is at least 95%. Furthermore, a positive interdependence in the higher order relationship between RTs and TAs is found. This implies that on average, test-takers who work overall faster also lose less time before making the first move in the item solving process. The results indicate that it is necessary to model the implied covariance structure of the correlated person effects on each type of test-taker data (RAs, RTs, and TAs). The variation in the data explained on a person level that is captured by the latent factors (ability, working speed, and speed first action) and their correlation is estimated through the corresponding layers in the additive covariance structure: modeling the person effects themselves is not required.

Neither variations in the item domains, nor in the response modes caused local dependence in the data. For each domain and response mode, three sources of local dependence are independently evaluated: the relationships between, respectively, (1) RAs and RTs, (2) RAs and TAs, and (3) RTs and TAs. Modeling the 3-by-3 covariances for each specified subset of items shows that the interdependences across data types and the investigated item characteristics are sufficiently captured by the covariance layers through which the dependences of the latent factors structure are specified. It can therefore be concluded that the items' domain and response mode do not explain a noticeable amount of variance in the test-taker data when controlling for the rest of the dependence structure.

The occurrence of a vast number of covariance parameter estimates close to, or approximately equal to, zero highlights the importance of the truncated shifted inverse-gamma prior specification that avoids boundary effects by moving the edge of the parameter space away from zero. For instance, a default inverse-gamma prior would presume that θ_20_>0 and would therefore be informative with regard to the probability of local dependence caused by the cross-relationship of RAs and TAs that belong to items with the stimulus clicking response mode: it decreases the estimated probability of local independence, i.e., the (estimated) probability that the true value of θ_20_ is zero, and can thereby provoke false conclusions about the underlying response processes. Finally, measurement error variance parameters are estimated for the fifteen RT components (mean: 0.62, SD: 0.47) and the fifteen TA components (mean: 0.31, SD: 0.19).

## 7. Discussion

A novel Bayesian framework to model local dependences in test-taker data is proposed. The BCSM allows specifying dependences across different types of data (RAs, RTs and other process data) and multiple levels (e.g., within a testlet, clustered data per item and test-taker). The local dependences are specified through a cross-classification structure and are explicitly modeled as covariance parameters. In an additive covariance structure, nested and/or cross-classified data structures are modeled through covariance parameters.

Recording test-taker data during CBAs is not limited to scored responses and RTs. For researchers and assessors these additional process data are of utility: they can increase the precision of test-taker ability estimates and lend new insights into underlying response processes. However, using process data to draw inferences is problematic in the GLMM framework: each additional type of data requires the inclusion of new person-level variables. If interaction effects occur, the model's complexity further increases drastically. A highly complex model is prone to over-parameterization and weak numerical stability, which may strongly limit its utility in practical applications.

The BCSM framework allows the construction of parsimonious models without requiring random effects (on a person or group level) to model data dependences. Contrary to common marginal modeling approaches such as GEE, the dependence structure is however fully modeled in an additive covariance structure. This allow testing for interaction effects and to estimate the random effects *post-hoc* from the residuals of the model. By estimating random effects *post-hoc*, inferences about test-taker characteristics can be made conditional on a complex within-subject dependence structure that follows from combining various auxiliary process data types into a coherent model. There is no theoretical limitation to the number of data types to combine, or in the number of components within each type (e.g., test length).

Modeling local dependences through covariance parameters instead of random effect variance parameters results in an extended parameter space. This allows more flexibility in specifying complex dependence structures (covariances can be negative, zero or positive). Compared to default inverse-gamma priors for variance parameters, truncated shifted inverse-gamma priors for the covariance parameters are less informative and allow more objective inferences about the dependence structure. The truncation is furthermore used to ensure the positive definiteness of the additive covariance structure, and can be utilized for inequality hypothesis testing (e.g., θ_1_ < θ_2_ < θ_3_). Through conjugacy of the proposed priors, BCSMs can be fit with an efficient Gibbs-sampling algorithm.

In a simulation study, a complex within-subject dependence structure was successfully estimated under a BCSM for responses and RTs. The model used for data generation allowed the test-takers' working speed and ability to vary over the course of a test. The LHM was not capable to capture this variation and showed bias in the variance estimate of the ability distribution. Under the BCSM, variation in test-takers' working speed and ability did not violate the condition of local independence: the dependence structure was extended to account for the variation. Furthermore, by estimating the extended dependence structure, insight into the development of the speed-accuracy trade-off on group level across the test was obtained.

The empirical example based on the PIAAC study showed a complex real-world dependence structure in response and process data. Covariance, measurement error variance and item parameters were estimated conditional on a dependence structure that took into account the classifications across three data types (scored dichotomous responses, RTs, TAs), item characteristics (domain, response mode), and the latent factor structure (data components load on the correlated factors ability, working speed and speed first action). Furthermore, test-taker background variables were included as covariates to correct for between-subject differences in the dependence structure. Through additive layers in a single covariance matrix, 3-by-3 covariance structures were modeled for each specified subset of items. This allowed to evaluate the cross-dependence between all pairs of data types individually for each of the item domains and response modes. The results indicated, that the interdependences across data types and the investigated item characteristics were sufficiently captured by the covariance layers through which the dependences of the latent factors structure were specified. The empirical example illustrates how, in the BCSM framework, the modeled dependence structure can be flexibly adapted to the design and the underlying theoretical constructs of an assessment. Furthermore, the vague nature of the truncated shifted inverse-gamma prior specification promotes unbiased inferences about the dependence structure. In the empirical example, this was in particular important due to the vast number of covariance parameter estimates close to, or approximately equal to zero. In this situation, a prior specification that does not take boundary effects into account artificially increases the estimated probability of local independence and hence provokes false conclusions about the dependence structure and the underlying response processes.

In addition to integrating multiple types of test-taker data, dependences can follow from the test design, item properties, the (sub-)population of test-takers, test-taking modes, test-taking conditions, and from an interaction of these characteristics. Examples are testlet structures, in which data within a testlet is often more alike then data across testlets (e.g., Wainer and Kiely, 1987; Yen, 1993; Wainer et al., 2007), or the interaction of culturally loaded concepts in items and diverse (sub-)populations of test-takers (e.g., with and without migration background) (e.g., Steele and Aronson, 1995; Paniagua, 2000; Good et al., 2003; Robinson, 2010). The resulting dependences in test-taker data form a threat for the flawless psychometric equivalence of an assessment, if not accounted for Helms (1992).

In educational measurement, factor loadings, or slope parameters, are utilized to assess differential item functioning (DIF) across groups, test-taking modes and over time (Millsap, 2010), allow multidimensional item response theory (MIRT) (Reckase, 2009), and are used to represent the quality of an item to discriminate between distributions of test-takers with a different level of ability or speed (van der Linden, 2007; Klein Entink et al., 2008). As discussed by Klotzke and Fox (2018), factor loadings integrate seamlessly into the proposed modeling framework. In fact, the inclusion of factor loadings solely removes the restriction of values being either zero or one in the classification matrix, hence keeping the modeling structure and the therein derived equations intact. However, while this allows to include pre-calibrated factor loadings into the model, no estimation procedure has been described so far. In a conditional-BCSM hybrid model, the factor loadings can also be modeled in the mean structure instead of in the covariance structure. For example, a 2PL-IRT model with item-discrimination parameters can be specified in the mean structure and the dependences implied by a testlet structure can be explicitly modeled in the multivariate distribution of the error terms. This approach is straightforward and suited for practical applications. A downside is, that a trade-off is been made between the parsimony of the model and the number of person level variables included in the mean structure. In the empirical PIAAC data example showcased, the factor loadings were predefined given the test design and item characteristics. Freeing the factor loadings will further increase the flexibility in the modeled dependence structure and thereby the utility of BCSM for practical applications in educational measurement.

It has been shown that modeling a non-linear relationship between RAs and RTs can be beneficial (e.g., Molenaar et al., 2015; Bolsinova and Molenaar, 2018). Through the additive covariance structure in BCSM, the conditional dependence between RTs and RAs is not limited to vary solely based on item membership (i.e., data points that belong to the same item are conditionally more alike), but is allowed to change based on item characteristics (e.g., domain and response mode), test form (e.g., computer based vs. paper-and-pencil) and test design (e.g., a testlet structure). Individual test-taker characteristics that may cause between-subject differences in the dependences of RTs and RAs are controlled for through modeling test-taker background variables as covariates in the mean structure (e.g., the relationship between RTs and RAs may vary based on the test-takers' age or a pre-test speed categorization). This differs from methods that model a non-linear relationship between RTs and RAs through a predefined function that involves person-specific random components and/or item parameters (Molenaar et al., 2015; Bolsinova et al., 2017; Bolsinova and Molenaar, 2018): in BCSM, test-taker characteristics that may affect the relationship between data types are controlled for in the mean structure, and the person-specific random effects are not modeled. Item characteristics are modeled in the mean structure (e.g., item difficulty parameters) and through additive layers in the covariance structure (e.g., item response mode). It is an interesting future prospect to see in how far the BCSM framework can be extended for covariance structures that follow from curvilinear functions for the relationship between data types. Furthermore, the BCSM approach must be distinguished from methods that model a person-specific covariance matrix (e.g., Meng et al., 2015): by their nature, models that explicitly specify a covariance matrix for each test-taker heavily increase in complexity with growing sample size and thus must impose strong restrictions on the modeled dependence structure to achieve model identification. In contrast, BCSM aims at designing parsimonious models that are easily identified when complex dependence structures are modeled.

The BCSM framework is not limited to RTs and dichotomous responses. Dependences between dichotomous responses and RTs were modeled through latent continuous variables. Expressions for the mean and variance of the conditional normal distribution of a latent variable were obtained by partitioning the additive covariance matrix and analytically deriving its inverse. Information from the observed responses (whether or not a test-taker responded correctly to an item) was utilized by truncating the respective distribution. Modeling dependences through latent continuous variables can be extended to data with more than two ordered or unordered response categories (e.g., Castro et al., 2012). This extends the range of process data that can be integrated into a BCSM. For example, sequential action patterns can be operationalized as count variables through N-grams (He et al., 2016). It is interesting to see under which conditions a BCSM allows to draw inferences about the interdependence between responses, RTs and action patterns and which new insights into latent response processes can be obtained. Further future prospects of BCSMs are the application to additional real-world empirical settings, extensions to unbalanced data and nested classifications on a person level (e.g., a test-taker is part of a school and classroom), and evaluating the utility of estimating test-taker effects *post-hoc*. Finally, it is of interest to compare the plausibility of different dependence structures in a Bayesian model selection framework (e.g., Kass and Raftery, 1995).

## Author Contributions

KK wrote the manuscript, developed the software and performed the analysis. J-PF and KK developed the modeling framework and the MCMC algorithm. J-PF contributed to the structure of the manuscript, and gave suggestions for the analysis and writing.

### Conflict of Interest Statement

The authors declare that the research was conducted in the absence of any commercial or financial relationships that could be construed as a potential conflict of interest.
