# Supplementary material for: Bayesian Covariance Structure Modeling of Responses and Process Data
Source: Front Psychol. 2019 Aug 5;10:1675. doi: 10.3389/fpsyg.2019.01675 (PMC6690231; doi:10.3389/fpsyg.2019.01675)
Supplement: Supplementary file 1 [file Data_Sheet_1.pdf]

Appendix A

Gibbs-Sampling Algorithm

`/* Initialize */`

Initialize MCMC chain with starting values

for *2:Iterations* **do**

> `/* Response accuracy */`
>
> Sample response accuracies $\boldsymbol{RA}|\boldsymbol{D}, \boldsymbol{RT}, \boldsymbol{u}, \boldsymbol{X}, \boldsymbol{B}^{m-1}, \boldsymbol{\theta}^{m-1}, \boldsymbol{\sigma}^{2^{m-1}}$
>
> `/* Mean structure */`
>
> Sample regression weights $\boldsymbol{B}|\boldsymbol{RT}, \boldsymbol{RA}, \boldsymbol{u}, \boldsymbol{X}, \boldsymbol{\theta}^{m-1}, \boldsymbol{\sigma}^{2^{m-1}}$
>
> `/* Covariance structure */`
>
> Sample measurement error variance parameters $\boldsymbol{\sigma}^2|\boldsymbol{RT}, \boldsymbol{u}, \boldsymbol{X}, \boldsymbol{B}, \boldsymbol{\theta}^{m-1}$
>
> Sample covariance parameters $\boldsymbol{\theta}|\boldsymbol{RT}, \boldsymbol{RA}, \boldsymbol{u}, \boldsymbol{X}, \boldsymbol{B}, \boldsymbol{\sigma}^2$

end

`/* Summarize */`

Compute posterior mean estimates from MCMC samples

Sampling steps

1. **Sample response accuracies**

$$RA_{ik} \sim Truncated - Normal\left(E\left(RA_{ik}|\cdot\right), Var\left(RA_{ik}|\cdot\right)\right),$$

where $E\left(RA_{ik}|\cdot\right)$ and $Var\left(RA_{ik}|\cdot\right)$ follow from Equation 3 and 4. For a correct response ($D_{ik} = 1$), the RAs (latent responses) are sampled from the interval $[0, \infty)$. Given an incorrect response ($D_{ik} = 0$), sampling is limited to the complementary interval $(-\infty, 0]$.

2. **Sample regression weights**

$$\boldsymbol{B} \sim \mathcal{MN}\left(\boldsymbol{\Upsilon}, \boldsymbol{\Omega}^{-1}, \boldsymbol{\Sigma}\right),$$
$$\boldsymbol{\Upsilon} = \boldsymbol{\Omega}^{-1}\left(\boldsymbol{X}^T\boldsymbol{Y} + \boldsymbol{\Omega}_0\boldsymbol{\Upsilon}_0\right),$$
$$\boldsymbol{\Omega} = \boldsymbol{X}^T\boldsymbol{X} + \boldsymbol{\Omega}_0,$$

where $\boldsymbol{\Upsilon}_0$ is a $N_X \times N_c$-dimensional matrix of zeroes, $\boldsymbol{\Omega}_0$ is the $N_X \times N_X$-dimensional identity matrix, N_X equals the number of predictors plus one, and $\boldsymbol{Y} = \{\boldsymbol{RT}, \boldsymbol{RA}\}$. $\mathcal{MN}$ denotes the Matrix Normal distribution (Gupta & Nagar, 1999, Chapter 2).

3. **Sample measurement error variance parameters**

$$\sigma_k^2 \sim IG(x, \alpha_0 + N/2, \beta_0 + SSW_k/2, \sum_{t=1}^{N_t} \theta_t u_{tk}, 0),$$

where $SSW_k = \sum_{i=1}^{N}\left(Y_{ik}^* - \bar{Y}_{.k}^*\right)^2$, $\boldsymbol{Y}^* = \boldsymbol{Y} - \boldsymbol{XB}$, and $\boldsymbol{Y} = \{\boldsymbol{RT}, \boldsymbol{RA}\}$.

4. **Sample covariance parameters**

$$\theta_t \sim IG(x, \alpha_0 + N/2, \beta_0 + SSB_t/2, \psi_t, tr_t),$$

where $SSB_t = \sum_{i=1}^{N}\left(\bar{Y}_{i(k \in \boldsymbol{u}_t)}^* - \bar{Y}_{.(k \in \boldsymbol{u}_t)}^*\right)^2$, $\boldsymbol{Y}^* = \boldsymbol{Y} - \boldsymbol{XB}$, and $\boldsymbol{Y} = \{\boldsymbol{RT}, \boldsymbol{RA}\}$. The truncation point ($tr_t$) and shift parameter ($\psi_t$) are computed according to Equation 6 and 7.

Appendix B

Classification Matrix $\boldsymbol{u}$

Response Accuracies															Response Times															Times to First Action														
1	1	1	1	1	1	1	1	1	1	1	1	1	1	1	0	0	0	0	0	0	0	0	0	0	0	0	0	0	0	0	0	0	0	0	0	0	0	0	0	0	0	0	0	0
0	0	0	0	0	0	0	0	0	0	0	0	0	0	0	1	1	1	1	1	1	1	1	1	1	1	1	1	1	1	0	0	0	0	0	0	0	0	0	0	0	0	0	0	0
0	0	0	0	0	0	0	0	0	0	0	0	0	0	0	0	0	0	0	0	0	0	0	0	0	0	0	0	0	0	1	1	1	1	1	1	1	1	1	1	1	1	1	1	1
1	1	1	1	1	1	1	1	1	1	1	1	1	1	1	1	1	1	1	1	1	1	1	1	1	1	1	1	1	1	0	0	0	0	0	0	0	0	0	0	0	0	0	0	0
1	1	1	1	1	1	1	1	1	1	1	1	1	1	1	0	0	0	0	0	0	0	0	0	0	0	0	0	0	0	1	1	1	1	1	1	1	1	1	1	1	1	1	1	1
0	0	0	0	0	0	0	0	0	0	0	0	0	0	0	1	1	1	1	1	1	1	1	1	1	1	1	1	1	1	1	1	1	1	1	1	1	1	1	1	1	1	1	1	1
1	1	1	1	1	1	1	1	1	0	0	0	0	0	0	1	1	1	1	1	1	1	1	1	0	0	0	0	0	0	0	0	0	0	0	0	0	0	0	0	0	0	0	0	0
1	1	1	1	1	1	1	1	1	0	0	0	0	0	0	0	0	0	0	0	0	0	0	0	0	0	0	0	0	0	1	1	1	1	1	1	1	1	1	0	0	0	0	0	0
0	0	0	0	0	0	0	0	0	0	0	0	0	0	0	1	1	1	1	1	1	1	1	1	0	0	0	0	0	0	1	1	1	1	1	1	1	1	1	0	0	0	0	0	0
0	0	0	0	0	0	0	0	0	1	1	1	1	1	1	0	0	0	0	0	0	0	0	0	1	1	1	1	1	1	0	0	0	0	0	0	0	0	0	0	0	0	0	0	0
0	0	0	0	0	0	0	0	0	1	1	1	1	1	1	0	0	0	0	0	0	0	0	0	0	0	0	0	0	0	0	0	0	0	0	0	0	0	0	1	1	1	1	1	1
0	0	0	0	0	0	0	0	0	0	0	0	0	0	0	0	0	0	0	0	0	0	0	0	1	1	1	1	1	1	0	0	0	0	0	0	0	0	0	1	1	1	1	1	1
0	0	0	0	0	1	0	1	0	0	0	0	0	0	0	0	0	0	0	0	1	0	1	0	0	0	0	0	0	0	0	0	0	0	0	0	0	0	0	0	0	0	0	0	0
0	0	0	0	0	1	0	1	0	0	0	0	0	0	0	0	0	0	0	0	0	0	0	0	0	0	0	0	0	0	0	0	0	0	0	1	0	1	0	0	0	0	0	0	0
0	0	0	0	0	0	0	0	0	0	0	0	0	0	0	0	0	0	0	0	1	0	1	0	0	0	0	0	0	0	0	0	0	0	0	1	0	1	0	0	0	0	0	0	0
1	0	1	1	0	0	1	0	0	0	0	0	0	0	0	1	0	1	1	0	0	1	0	0	0	0	0	0	0	0	0	0	0	0	0	0	0	0	0	0	0	0	0	0	0
1	0	1	1	0	0	1	0	0	0	0	0	0	0	0	0	0	0	0	0	0	0	0	0	0	0	0	0	0	0	1	0	1	1	0	0	1	0	0	0	0	0	0	0	0
0	0	0	0	0	0	0	0	0	0	0	0	0	0	0	1	0	1	1	0	0	1	0	0	0	0	0	0	0	0	1	0	1	1	0	0	1	0	0	0	0	0	0	0	0
0	1	0	0	1	0	0	0	1	1	0	1	1	0	0	0	1	0	0	1	0	0	0	1	1	0	1	1	0	0	0	0	0	0	0	0	0	0	0	0	0	0	0	0	0
0	1	0	0	1	0	0	0	1	1	0	1	1	0	0	0	0	0	0	0	0	0	0	0	0	0	0	0	0	0	0	1	0	0	1	0	0	0	1	1	0	1	1	0	0
0	0	0	0	0	0	0	0	0	0	0	0	0	0	0	0	1	0	0	1	0	0	0	1	1	0	1	1	0	0	0	1	0	0	1	0	0	0	1	1	0	1	1	0	0
0	0	0	0	0	0	0	0	0	0	1	0	0	1	1	0	0	0	0	0	0	0	0	0	0	1	0	0	1	1	0	0	0	0	0	0	0	0	0	0	0	0	0	0	0
0	0	0	0	0	0	0	0	0	0	1	0	0	1	1	0	0	0	0	0	0	0	0	0	0	0	0	0	0	0	0	0	0	0	0	0	0	0	0	0	1	0	0	1	1
0	0	0	0	0	0	0	0	0	0	0	0	0	0	0	0	0	0	0	0	0	0	0	0	0	1	0	0	1	1	0	0	0	0	0	0	0	0	0	0	1	0	0	1	1

Figure 4: Table 1: classification matrix for the PIAAC BCSM. Classifications are made across three data types (scored dichotomous responses, RTs, times to first action taken) based on correlated underlying latent factors (ability, working speed, speed first action), test-taker membership, and item characteristics (numeracy items, literacy items, response mode).
